# The assembly of caprine Y chromosome sequence reveals a unique paternal phylogenetic pattern and improves our understanding of the origin of domestic goat

**DOI:** 10.1002/ece3.7611

**Published:** 2021-05-04

**Authors:** Changyi Xiao, Jingjin Li, Tanghui Xie, Jianhai Chen, Sijia Zhang, Salma Hassan Elaksher, Fan Jiang, Yaoxin Jiang, Lu Zhang, Wei Zhang, Yue Xiang, Zhenyang Wu, Shuhong Zhao, Xiaoyong Du

**Affiliations:** ^1^ College of Informatics Huazhong Agricultural University Wuhan China; ^2^ Key Laboratory of Agricultural Animal Genetics, Breeding and Reproduction Ministry of Education College of Animal Science and Veterinary Medicine Huazhong Agricultural University Wuhan China; ^3^ Institutes for Systems Genetics Frontiers Science Center for Disease‐related Molecular Network West China Hospital Sichuan University Chengdu China; ^4^ Genetics and Genetic Engineering Department Faculty of Agriculture Benha University Moshtohor Egypt; ^5^ College of Agroforestry Engineering and Planning Tongren University Tongren China

**Keywords:** demographic history, goat, phylogenetic, RH map, Y chromosome assembly

## Abstract

The mammalian Y chromosome offers a unique perspective on the male reproduction and paternal evolutionary histories. However, further understanding of the Y chromosome biology for most mammals is hindered by the lack of a Y chromosome assembly. This study presents an integrated in silico strategy for identifying and assembling the goat Y‐linked scaffolds using existing data. A total of 11.5 Mb Y‐linked sequences were clustered into 33 scaffolds, and 187 protein‐coding genes were annotated. We also identified high abundance of repetitive elements. A 5.84 Mb subset was further ordered into an assembly with the evidence from the goat radiation hybrid map (RH map). The existing whole‐genome resequencing data of 96 goats (worldwide distribution) were utilized to exploit the paternal relationships among bezoars and domestic goats. Goat paternal lineages were clearly divided into two clades (Y1 and Y2), predating the goat domestication. Demographic history analyses indicated that maternal lineages experienced a bottleneck effect around 2,000 YBP (years before present), after which goats belonging to the A haplogroup spread worldwide from the Near East. As opposed to this, paternal lineages experienced a population decline around the 10,000 YBP. The evidence from the Y chromosome suggests that male goats were not affected by the A haplogroup worldwide transmission, which implies sexually unbalanced contribution to the goat trade and population expansion in post‐Neolithic period.

## Graphical abstract

In this paper, we first assembled goat Y chromosome into 33 scaffolds totaling 11.5 Mb, and a subset of 5.84 Mb was further ordered into an assembly with the validation of goat radiation hybrid map. Then, we identified SNPs of 96 goat samples worldwide to exploit the paternal relationships among wild bezoars and domestic goats. The paternal lineages showed a clear division of bezoars and goats into two clades, and they were independent of goat domestication. Further demographical history of paternal and maternal lineages showed evident differences, with a bottleneck effect in mtDNA around 2,000 YBP, which may be related to the worldwide dispersal of goat in the post‐Neolithic period, implied the possibility of gender preference in goat domestication. Our research provided a new perspective of goat evolution history.

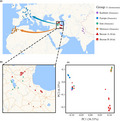

## 1 INTRODUCTION

The Y chromosome sequence is essential for studies of male reproductive traits (Case & Teuscher, 2015), chromosomal evolutionary history (Skaletsky et al., 2003), and patrilineal phylogeographic distribution (Karmin et al., 2015; Mendez et al., 2011). The current Y chromosome's gene content and organization have evolved from a homologous pair of autosomes through isolation and accumulation of male‐beneficial genes, generally involved in spermatogenesis (Cortez et al., 2014; Livernois et al., 2012). Unlike autosomes, the crossing over between the male‐specific region of the Y chromosome (MSY) and X chromosome is inhibited due to a series of inversions on the Y chromosome (Skaletsky et al., 2003). This implies that Y chromosome sequence cannot evolve via recombination, but only via mutations. Furthermore, the fact that MSY can only be inherited from father to son allows us to trace the paternal history, reflecting unique paternal migrations and demographic histories, relatively accurately over long periods of time (Semino et al., 2000; Underhill et al., 2000). For example, recent phylogenetic research on horses and wolves found different demographic histories between males and females (Smeds et al., 2019; Wallner et al., 2017). Therefore, tracing the demographic histories of the two sexes independently can help us better understand the evolutionary process of a species.

However, due to the haploid nature of Y chromosome, shot‐gun genome‐sequencing process produces only half the sequencing depth compared to autosomes. Due to this problem, specimens homozygous for sex chromosomes are often chosen for genome assemblies to get sufficient coverage of the sex (X or Z) chromosome (Rangavittal et al., 2018). Furthermore, most of the Y chromosome sequence is constitutive heterochromatin, which consists of a large number of repetitive elements (Iannuzzi & Di Meo, 1995). This additionally hampers sequencing and assembly efforts. As a result of these factors, only a limited number of mammalian genomes have a full Y assembly. This relative scarcity of Y assemblies hampers studies of the evolution of Y chromosome in mammals.

Previously, several different methodological approaches were used to assemble Y chromosome. BAC (bacteria artificial chromosome) clones were used for sequencing and scaffolding for human (Skaletsky et al., 2003), mouse (Soh et al., 2014), rhesus macaque (Hughes et al., 2012), dog, cat (Li et al., 2013), and horse (Hughes et al., 2012). For chimpanzee (Hughes et al., 2010), flow cytometry separation followed by a single‐molecule sequencing was used. For polar bear (Bidon et al., 2015) and gorilla (Tomaszkiewicz et al., 2016), bioinformatic approaches based on the difference of male–female sequence coverage or kmer distribution were utilized (Bidon et al., 2015; Carvalho & Clark, 2013; Tomaszkiewicz et al., 2016). Previous efforts have generated several goat genome assemblies: CHIR_1.0, ARS1, CVASU_BBG_1.0, and ASM982349v1. CHIR_1.0 version was the first goat genome assembly using paired‐end short reads from a female Yunnan black goat, with a genome size of 2.66 Gb and a scaffold N50 length of 3.06 Mb (Dong et al., 2013). The ARS1 goat genome effectively improved the continuity and the accuracy of the genome assembly using the combination of long‐read single‐molecule sequencing, short‐read sequencing, Hi‐C data, and optical mapping data from a male San Clemente goat, with N50 length of 18.7 Mb and genome size of 2.9 Gb (Bickhart et al., 2017). CVASU_BBG_1.0 (Siddiki et al., 2019) and ASM982349v1 are assemblies of a Bengali goat and a Kashmiri goat, respectively; these two assemblies produced comparatively larger N50 sizes. Despite these multiple sequences, only ten Y‐linked scaffolds have been identified using the homology of cattle Y‐linked genes or microsatellites (Bickhart et al., 2017), so the landscape of the goat Y chromosome remains only partially known.

Domestication of the goat during the Neolithic period was one of the most advanced achievements for early agricultural societies, for which goats provided a stable source of meat, milk, and fiber. Both archeological and genetic evidence indicates that the modern domestic goat (*Capra hircus*) was initially domesticated in the Fertile Crescent from the bezoar (*Capra aegagrus*), with a possible minor contribution of other *Capra* species (*Capra falconeri, Capra pyrenaica,* and *Capra ibex*, etc.) (Daly et al., 2018; Fernandez et al., 2006; Naderi et al., 2008; Zeder & Hesse, 2000). Over the 10,000 ~ 10,500 years, domestic goats were gradually dispersed into other parts of the world (Amills et al., 2017). Most goat phylogeographic studies focused on mtDNA data (Kamalakkannan et al., 2018; Naderi et al., 2008) and autosomal data (Bertolini et al., 2018; Kumar, Song et al., 2018; Zheng et al., 2020), whereas phylogeographic studies on the Y chromosome are relatively few. Most of these studies only focused on sequences of a few Y‐linked genes (*SRY*, *AMELY*, and *ZFY*) or parts of the male‐specific region on the Y chromosome (Cinar Kul et al., 2015; Nijman et al., 2020; Pidancier et al., 2006; Tabata et al., 2018; Vidal et al., 2017; Waki et al., 2015). The limited phylogenetic signal carried by the Y chromosome fragments may not be powerful enough to give sufficient independent and complementary information for closely related breeds in paternal dispersal analysis (Boissinot & Boursot, 1997).

Using existing data for new bioinformatics analyses is an economical way to conduct scientific research, and it can help us fully exploit the existing data resources. In this study, we aimed to (a) better classify the Y‐linked sequences through integrated bioinformatics approaches using unplaced sequences from ARS1; (b) order and assemble scaffolds by chromatin interaction information and radiation hybrid map (RH map); (c) annotate this Y assembly including repetitive elements and protein‐coding genes; (d) reveal the main paternal goat origins based on haplogroups of Y sequence. In summary, our results clustered 33 Y‐linked scaffolds with a total size of 11.5 Mb, of which a 5.84 Mb subset was further ordered with the validation of the RH map. The subsequent phylogenetic analysis using these scaffolds and global data for domestic goats and bezoars revealed possible paternal lineages in the caprine history and evolution.

## 2 METHODS

### 2.1 Identification of Y‐linked scaffolds

As sex information was not recorded for some samples available in public database, a bioinformatics approach based on detecting the coverage depth was developed to efficiently identify the sex for each sequenced goat sample. We mapped the sequence data of ten known male and ten known female samples to one scaffold containing the *SRY* gene using Bowtie (v1.3.0) (Langmead et al., 2009) with default parameters. We then calculated the difference of coverage between sexed samples in this scaffold using SAMtools (v1.3) (Li et al., 2009) with the parameter “‐aa.” Based on the statistics, a batch of sequences totaling 268 Kb was truncated to represent male seed sequences. The seed sequences were indexed in Bowtie as a reference genome to calculate the mapping rate of each unrecorded sample and identify their sex. We then mapped ten males and ten females to the seed sequences and counted their mapping rate. The results found that the mapping rate of the male sample was greater than 0.04%, while the female sample was 0.00% (Table S1) and t test statistics were significant (*p* = 8.65e^−8^). Thus, these two values were designated as thresholds to identify sex.

Samples with mapping rate between these two values were ambiguous in sex and were excluded from our study.

The unplaced scaffolds of ARS1 were used for the assembly of the goat Y chromosome. Three approaches were used in this study—the YGS approach, the AD ratio approach, and the similarity search.

1. The YGS approach was originally used to identify nearly all Y chromosome sequence in the human and *Drosophila* genomes with high accuracy (Carvalho & Clark, 2013). In this method, Y‐linked sequences can be identified by comparing the scaffolds with a dictionary database of short sequences obtained from female whole‐genome DNA. The Y‐linked sequences should get no match, whereas autosomal and X‐linked sequences should be nearly completely matched. This method has the highest precision in identifying Y‐linked sequences, but the existence of SNPs can reduce its precision. With the length of kmer set to 31 bp, we classified reads that matched with male short reads at a rate larger than 90%, and less than 10% with female short reads, as Y‐linked scaffolds. Short reads of five male goats and ten female goats from the NextGen project (BioProject PRJEB3135, Table S2) were used to build a dictionary of sequences of kmer = 31 for males and females. All queried nonrepetitive sequences of kmer = 31 from scaffolds were compared with the dictionary database using Jellyfish (v2.2.6) (Marcais & Kingsford, 2011).
2. The AD ratio method was originally applied to identify Y‐linked sequences of polar bears (Bidon et al., 2015). As unique Y chromosomal sequences are not present in the female genome, short reads of female and male individuals should map with characteristic sex‐specific patterns to scaffolds from the Y chromosome, the X chromosome, and the autosomes. This method has higher tolerances to SNPs and haplotypes than the YGS method. AD ratio of each scaffold was calculated by dividing the average read depth in the female individual to male individual following the formulas:

AD ratio = average‐depth_female_/(average‐depth_male_ × norm)

norm = total number of reads_female_/total number of reads_male_

In this study, short reads of five male goats and five female goats (both from NextGen project PRJEB3135, Table S2) were used to calculate AD ratios. One female goat reference (GenBank assembly accession: GCA_000317765.2) was added to the unplaced scaffolds when building the BWA (v0.7.12) index files (Li, 2013) to reduce the effects of mismatch caused by small size of scaffolds. BWA was used to align the reads with default parameters, and SAMtools was used to calculate the coverage depth of each locus. AD ratio for each scaffold was calculated using our own script. Ten previously identified Y chromosomal scaffolds (Bickhart et al., 2017) with AD ratios <0.3 were used as a positive control (Table S3). Since the Y chromosome is degenerated from the X chromosome, it has some similarities with the X chromosome. For example, the X‐transposed regions on the human Y chromosome exhibit 99% identity to the X chromosome, making it possible that female reads are also mapped on the Y chromosome. Thus, we applied a relatively relaxed cutoff AD ratio <0.4 to minimize the chance of missing large Y‐linked scaffolds.

- For similarity search, we used the same approach and parameters as described in Bickhart et al. (2017). In our study, the autosomes and X chromosome were from ARS1 (PRJNA290100 and PRJNA340281, Table S2), and the version of the cattle Y chromosome was bt_alt_Btau_5.0.1_chrY.

### 2.2 Scaffolding using mate‐pair data and Hi‐C data

All Y‐linked sequences detected by the above three methods were combined, and then, we used mate‐pair data and Hi‐C data to extend and scaffold them. Firstly, reads from 21 mate‐pair libraries (SRP047212, PRJEB3140, and PRJNA290100, Table S2), whose insert size ranged from 500 bp to 40,000 bp, were trimmed using platanus_internal_trim (v1.0.7) (Kajitani et al., 2014) with default parameters. Then, we followed the standard OPERA‐LG (Gao et al., 2016) pipeline to scaffold Y‐linked scaffolds. BESST (v2.2.8) (Sahlin et al., 2014) was also used with default parameters to compare with OPERA‐LG results. The result showed that OPERA‐LG is more suitable for scaffolding highly repetitive sequences (Tables S4 and S5).

Secondly, reads carrying chromatin conformation information were used to assemble these scaffolds further. Arima Genomics (https://github.com/ArimaGenomics) was used to postprocess the alignments to counteract potential experimental artifacts before scaffolding. After this, SALSA (v2.2) (Ghurye et al., 2017; Zou et al., 2016) was used for scaffolding of our assembly with the restriction enzyme interaction frequency normalized to HindIII.

### 2.3 Ordering and orienting of scaffolds using RH map

Thirteen scaffolds (>200 Kb) were ordered and oriented by RH mapping. For each scaffold, 20 pairs of primers were designed in three regions (head, middle, and tail) for scaffolds larger than 1 Mb and two regions (head and tail) for scaffolds smaller than 1 Mb. During this process, RepeatMasker (v4.0.7) (Smit et al., 2013–2015) was used to mask known repeats to avoid false markers on repetitive sequences. Primer3 (v2.3.7) (Koressaar & Remm, 2007) was then used for primer designing with the optimal length of primer set to 21 bp (range from 18 to 24 bp) while the range of product size was from 650 to 850 bp. The sequence of each primer pair was first searched against ARS1 and all other unplaced scaffolds to ensure the primer's uniqueness using nucmer (v4.0.0) (Marçais et al., 2018) with the parameter “‐c 10 ‐l 10.” The goat RH panel includes 93 hybrid clones, one positive from a male goat (JEW105), one negative from a hamster cell line (A23), and blank control (water) (Du et al., 2012). For each primer pair, we had two PCR tests. If the electrophoresis test showed that some bands are not clear or strong enough, we would change for another pair of primer or a third PCR duplicate. All bands were recorded as present (H), absent (A), and ambiguous (?). We used 29 markers to generate RH vectors, of which 27 were assigned to two single linkage groups based on two‐point analysis (LOD threshold of 10); two markers did not show enough evidence for linkage. A map was first generated with Carthagene (Faraut et al., 2007; de Givry et al., 2004) by converting the RH data into a TSP (Traveling Salesman Problem) and solved using a LKH (Lin–Kernighan heuristic) method (lkh1 1) (Lin & Kernighan, 1973). RHMAPPER (Stewart et al., 1997) was used to flag marker/hybrid assay results that are likely to result from laboratory error with command find_errors, where the order for these results set to the primary alignment, and a threshold of 3 was specified (Du et al., 2014).

### 2.4 Repetitive elements annotation

Repeats were identified using the RepBase library (release 2014‐01‐31) with RepeatMasker. The “species” (goat, cattle) options were the only deviations from the default. Custom scripts were used to compare the distribution and class of repeats between the goat and cattle Y chromosome scaffolds.

### 2.5 Gene annotation

Three strategies were used to predict genes on all 33 Y‐linked scaffolds (>100 Kb): ab initio prediction, homology‐based annotation, RNA‐seq‐based annotation. We used GeneMark (v4.62) (Lomsadze et al., 2005) and AUGUSTUS (v3.3.2) (Stanke et al., 2006) to perform ab initio annotation. Exonerate (v2.4.0) (Slater & Birney, 2005) was used with the parameter “‐‐model protein2genome ‐‐percent 50” to perform homologous annotation. For RNA‐seq based annotation, two pipelines (a) Hisat2 (v2.0.0) + StringTie (v2.0.6) (Kim et al., 2015; Pertea et al., 2016) and (b) PASA (v2.3.3) + TransDecoder (v5.5.0) (Douglas, 2018; Haas et al., 2003) were both used to integrate the prediction with default parameters. Finally, EvidenceModeler (v1.1.1) (Haas et al., 2008) was used to merge results for predicted genes. Those predicted genes were aligned with the public NR database (Pruitt et al., 2005), with a threshold of E‐value <1e^−5^.

We only retained the annotation information related to mammals, and genes that met the following criteria were excluded: (a) the number of exons was no more than one; (b) the number of amino acids was less than 50; and (c) genes related to reverse transcriptase. After filtering, we obtained 187 high‐quality gene annotations.

### 2.6 SNP calling and variants annotation

We downloaded the whole‐genome resequencing data of 96 male individuals from several BioProjects in the EBI database (PRJEB3135, PRJEB3134, PRJEB26011, PRJNA310684, PRJNA422206, PRJNA514886, and PRJEB3136, Table S2) for variants calling and downstream analysis (Table S2). We combined the 33 Y‐linked scaffolds with the ARS1 autosomes and X chromosome genome to build index files by BWA, SAMtools, and Picard tools (v1.108) (Sentieon version). Known sites of SNPs were downloaded from the Ensembl as a standard model for SNP calling. A commercial GATK‐like pipeline, Sentieon, was used to call SNV variants for Y chromosome with the parameter “‐‐ploidy 1” and for autosomes with the parameter “‐‐ploidy 2” (Freed et al., 2017). SNV variants were first called in each sample separately with Sentieon's DNAscope algorithm. Then, joint genotyping was performed to merge all samples using Sentieon's GVCFtyper algorithm with the nearly same protocol in the “GATK Best Practices.” SNPs were hard‐filtered with bcftools using the settings “‐e ‘QD <2.0 || MQRankSum < −12.5 || FS >60.0 || ReadPosRankSum < −8.0 || MQ <40.0 || SOR >3.0’” (Van der Auwera et al., 2013). We also removed all SNPs close to structural variants (repeats, insertions, and deletions) called by Sentieon's SVSolve algorithm. To get high‐quality SNPs of the Y chromosome, we further called variants of 66 extra female samples (raw data from PRJNA310684, Table S2). Y chromosome SNPs found from these female samples were excluded to avoid ambiguous or incorrectly assembled regions. Then, we only kept biallelic SNPs with MAF ≥ 0.05 and call rate ≥90% using VCFtools (v0.1.15) (Danecek et al., 2011) and 6,914 Y chromosome SNPs out of 311,549 sites were kept for further phylogenetic analysis, of which a subset of 4,740 SNPs only exist in domestic goats and bezoars. For autosomes, we further excluded SNPs with *r* ^2^ ≥ 0.5 using Plink (v1.9) (Purcell et al., 2007) to avoid artifacts caused by linkage disequilibrium (LD). A total of 9,820,345 autosomal SNPs were kept.

The ORGanelleASeMbler software (http://pythonhosted.org/ORG.asm/) was used to de novo assemble the mitochondrial genomes from the whole‐genome paired‐end Illumina sequencing reads. We aligned these assembled mtDNA sequences with the reference mitochondrial sequence (NCBI accession number: NC_005044.2) and generated the consensus sequences.

### 2.7 Phylogenetic analyses

The SNPs of 33 Y‐linked scaffolds (>100 Kb) were used to reconstruct the phylogenetic relationships of 67 domestic goats, 24 bezoars, and five other *Capra* species sampled worldwide. We first performed PCA using GCTA (v1.92) (Yang, Lee et al., 2011) with and without outgroup samples. A total of 6,914 SNPs were then linked together to form sequences, which were then used to reconstruct the phylogenetic tree using the Yule process in BEAST (v1.10.4) (Gernhard et al., 2008; Suchard et al., 2018). The HKY+G substitution model was selected by comparing Bayesian information criterion (BIC) scores in jModelTest (v2.1.7) (Darriba et al., 2012), and a strict clock rate was used to construct the phylogenetic tree. The Bayesian phylogenetic analysis was run for 100,000,000 generations, with sampling every 5,000 generations, and 10% burn‐in. Convergence was checked in Tracer to make sure that ESS >225. We then used TreeAnnotator implemented in BEAST to summarize the MCMC samples as the maximum clade credibility topology. Full sequence data from scaffold_213 was used to generate haplotype data using DNASP (v6) (Rozas et al., 2017), which was further used to construct neighbor‐joining network using POPART (http://popart.otago.ac.nz) (Leigh & Bryant, 2015).

Autosomal SNPs were first used to construct a NJ tree with Plink using the matrix of pairwise genetic distances. Population genetic structure was inferred by the same dataset using ADMIXTURE (v1.3.0) (Alexander et al., 2009). The ADMIXTURE program was run in an unsupervised manner with a variable number of clusters (*K* = 2 to 6). For mtDNA, we first performed multisequence alignment using a muscle algorithm from MEGAX (v10.0.5) (Kumar, Stecher et al., 2018). Then, we used Gblocks (v0.91b) (Castresana, 2000; Talavera & Castresana, 2007) to extract conserved sites for NJ tree construction using MEGAX with Kimura 2‐parameter model.

The Bayesian skyline plot (BSP) was constructed using BEAST (Drummond et al., 2005) for 67 domestic goats using Y chromosome data and mtDNA sequence separately. Before estimating, we tested whether concatenate SNPs can replace the complete sequence for BSP analysis and whether the scaffolds' orders and orientations would affect the results. Therefore, concatenate SNPs from the Y chromosome were used for BSP analysis to reduce computational complexity. To estimate goat Y chromosome mutation rate, we used the mean goat genome mutation rate of 1.3e^−8^ per bp per generation (Daly et al., 2018), with generation time two years, and assumed a male‐to‐female mutation rate ratio of 2.0 (Smeds et al., 2019) (the inferred rate was 8.67e^−9^ for full sequence). Next, we rescaled the mutation rate into 7.14e^−6^ for concatenate SNPs (Table S6). Other parameters were the same as for the phylogenetic analysis. MCMC chain length of 100,000,000 was used to ensure ESS >225. The mtDNA model parameters were set under the HKY+G substitution model, and a strict clock rate with mutation rate of 2.73e^−7^ (Nomura et al., 2013; Tarekegn et al., 2018) was used for Bayesian skyline analysis.

## 3 RESULTS

### 3.1 The identification of Y‐linked scaffolds

**FIGURE 1:**
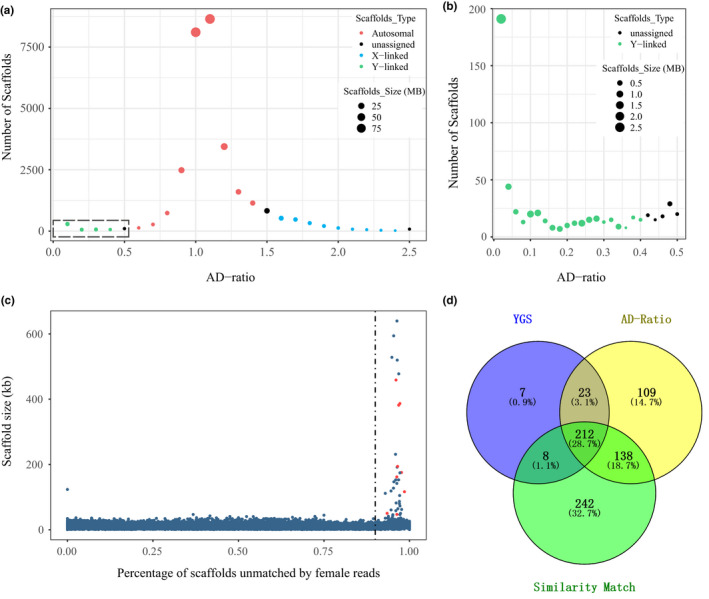
Identified scaffolds of the goat Y sequence. (a) AD ratios of X‐linked (blue), autosomal (red), Y‐linked (green), and unassigned scaffolds (black). (b) The enlargement of the box in (a), scaffolds below the threshold of 0.4 are classified as Y origin, and unassigned scaffolds are shown in black. (c) Identification of Y‐linked scaffolds through the YGS. Each dot represents one scaffold in all unplaced scaffolds. Red dots are scaffolds previously confirmed to be Y‐linked. Abscissa denotes the proportion of scaffold sequence not matched by female short reads; ordinate denotes scaffold size. The filtering cutoff was set to 90%. (d) The count of scaffolds identified by the AD ratio, the YGS, the similarity search, and the comparison of overlaps among all approaches

We performed integrated in silico strategies to identify Y‐linked scaffolds, and ten known Y chromosomal scaffolds (Bickhart et al., 2017) were used as the positive control. In the AD ratio algorithm, as unique Y chromosomal sequences are not present in the female genome, the coverage of reads from known female/male should exhibit distinct sex‐specific patterns on Y‐linked scaffolds. We categorized scaffolds into three types according to the value of AD: (a) scaffolds with AD ratio <0.4 (*n* = 482) corresponded to Y chromosomal origins; (b) scaffolds with 0.6 < AD ratio <1.4 (*n* = 26,560) were from autosomal chromosomes; (c) scaffolds with 1.6 AD ratio <2.4 (*n* = 1,857) were classified as X chromosomal sequences (Figure 1a). To further investigate the distribution of scaffolds with AD ratio <0.5, we plotted an enlarged view (Figure 1b). The accumulated length of these 482 Y‐linked scaffolds was 12.9 Mb with N50 length of 64.6 Kb.

In the YGS results (Figure 1c), the distribution is sharply unimodal. The right peak centering at 90%–100% unmatched of female kmer (N50 length = 146.9 Kb) corresponded to the Y chromosome, and ten positive control scaffolds were identified. The scaffolds below the cutoff value were of short length (N50 length = 13.5 Kb) and highly repetitive (82.13% could be masked and 67.81% of their content were satellites). The second approach identified 250 Y‐linked scaffolds (with the accumulated length of 11.0 Mb). Thirdly, we performed a similarity comparison between goat scaffolds and cattle Y sequence, and 600 goat Y‐linked scaffolds were identified with an accumulated size of 19.0 Mb.

In summary, we classified 212 scaffolds (totaling 9.6 Mb) from the intersection of three methods (Figure 1d), with a N50 size of 152.8 Kb, indicating the reliability of long scaffolds identified in this study. The entire union set of three approaches contained 739 Y‐linked scaffolds with an accumulated size of 20.8 Mb (N50 = 63.5 Kb), which were used for downstream extending and scaffolding.

### 3.2 Extending and validating the Y‐linked scaffolds

The assembly of repeat‐rich genome was always a major challenge for the current sequencing technologies. To extend scaffolds, reads from 21 mate‐pair libraries in male bezoars (Dong et al., 2015) and chromatin conformation information of Hi‐C in a male San Clemente goat (Bickhart et al., 2017) were used, and an improved assembly was generated (Table 1). The N50 size of the initial assembly extended by the mate‐pair long reads was 131.4 Kb, doubled from the original 63.5 Kb. Hi‐C data provided a relatively small contribution (N50 size of 132.2 Kb), of which 33 scaffolds were larger than 100 Kb, totaling 11.5 Mb (Figure S1).

**TABLE 1:** The statistics for Y‐linked scaffolds assembly

	Unplaced scaffolds		Y‐linked scaffolds		Scaffolding after MP		Scaffolding after Hi‐C	
	bp	Number	bp	Number	bp	Number	bp	Number
N10	48,493	205	519,723	4	639,497	3	1,171,813	2
N20	22,233	1,438	386,696	9	502,319	7	951,591	4
N30	18,100	3,160	231,175	15	313,531	13	525,554	6
N40	15,655	5,193	147,927	27	180,842	21	256,823	12
N50	13,520	7,543	63,513	48	131,477	34	132,205	24
N60	11,630	10,270	29,210	99	60,841	57	60,841	47
N70	9,880	13,448	21,420	184	30,800	107	32,117	96
N80	8,044	17,265	16,818	295	20,427	195	20,746	182
N90	6,244	22,066	12,043	440	13,217	321	13,239	307
Total	341,472,733	29,967	20,842,374	739	20,918,979	600	20,926,979	584

**FIGURE 2:**
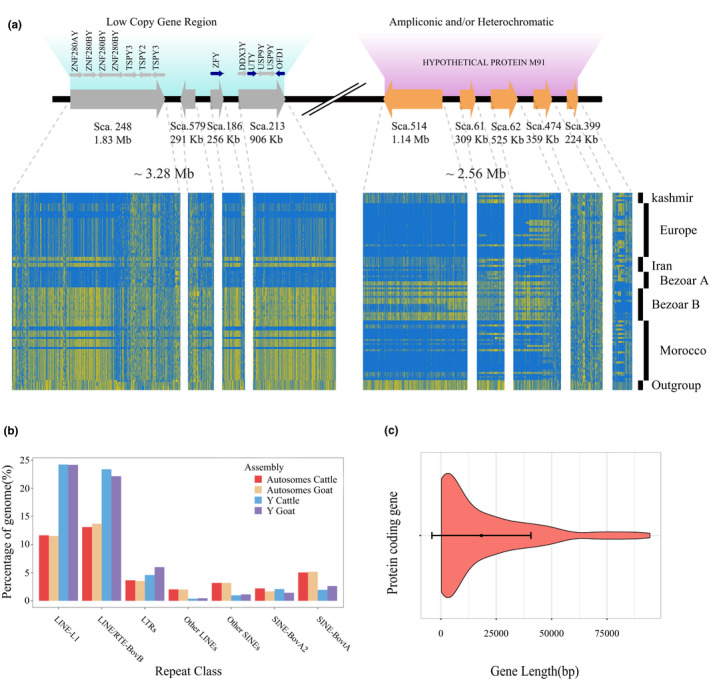
(a) The organization of the goat Y assembly. The assembly consists of two separate sections representing a low‐copy gene region and ampliconic and/or heterochromatic region. The single‐copy genes are blue, and the multicopy genes are gray. The haplotypes of 24 male bezoars, 67 male domestic goats, and five other male *Capra* species corresponding to the above organization are shown. The reference allele is blue and alternative allele is yellow. (b) The comparison of coverage of several major types of repetitive elements among the autosomes and Y sequences in cattle and goats. (c) The distribution and median size of protein‐coding genes

The goat RH panel from a male Boer goat was used to validate and orient these Y scaffolds (Du et al., 2012). This goat RH panel has been used to validate the goat genome assembly of CHIR_1.0 version and ARS1 (Bickhart et al., 2017; Dong et al., 2013, 2015). Based on the estimated resolution of 65 Kb in the RH panel, we only tested the longest 13 scaffolds (>200 Kb, totaling 8.7 Mb) in the panel of 93 RH clones, using 29 markers. We ordered and oriented nine scaffolds into two separated chromosomal sections (Table S7) with a length of 3.28 Mb and 2.56 Mb, separately (Figure 2a).

### 3.3 Genome annotation

The assembly of Y chromosome was organized into two blocks. The first block consisted of four scaffolds with an accumulated size of 3.28 Mb, and all identified male‐specific low‐copy genes are in this region. The second block harbored five scaffolds with a total of 2.56 Mb, and this region is highly ampliconic, with rich copies of one gene named hypothetical protein M91 (Figure 2a).

#### 3.3.1 High abundance of repetitive elements on the Y‐linked scaffolds

Compared with 45.77% of autosomal sequences covered with repeats, the repetitive content of Y‐linked scaffolds reached 56.66% in the goat genome. The most abundant repeat types were long interspersed nuclear elements (LINEs, 45.36%) and short interspersed nuclear elements (SINEs, 6.21%). Previous studies have revealed that repeats across autosomes exhibited ruminant‐specific patterns between cattle and goat (Adelson et al., 2009). The comparison showed that the Y assembly of goat and cattle had almost identical coverage of both LINEs and SINEs (Figure 2b). Yet, distinct distributions of repeats between Y and autosomal sequences were observed. For example, LINE‐L1 and LINE‐BovB exhibited much more extensive distribution than autosomal sequences, while SINE‐BovtA showed only half the coverage compared with autosomal genomes. These results suggest that the haploid nature of the Y chromosome may have caused a distinct environment for the evolution of retrotransposons compared with autosomes (Adelson et al., 2009; Charlesworth & Charlesworth, 2000).

We next analyzed the divergence for highly distributed retrotransposons in goat and cattle genomes (Figure S2). For LINEs, we noticed that BovB exhibited high coverage with low substitution levels, suggesting a recent expansion in cattle and goat. In contrast, L1 showed a bimodal distribution, which is likely a remnant of two ancient bursts of retrotransposon activities. For SINEs, both BovA2 and BovtA were unimodal with relatively lower divergence, which indicates that the burst of SINEs in the ruminant genome was much more recent than that of LINEs. Notably, we found that Y sequences harbored higher LINEs than autosomal sequences for both cattle and goat. This phenomenon may be caused by increased insertion of retrotransposons on Y chromosome due to the haploid Y nature, or massive gene decay during the Y evolution.

#### 3.3.2 Gene annotation

We identified 187 candidate protein‐coding genes totaling 3.4 Mb with a mean gene length of 18,315 bp in the goat Y chromosome (Figure 2c, Table S8). The average length of exons and introns was 186 bp and 5,238 bp, respectively. We then annotated these genes using the NR database. Three gene families were multicopy, and some genomic regions may have undergone major deletions in goat. In cattle, all these genes are functionally correlated with fertility and spermatogenesis:

(a) The *TSPY* family: *TSPY* (testis‐specific Y‐encoded protein 1‐like), *TSPY2*, and *TSPY3* are ampliconic gene families in many mammalian species, and copy number variations of this family have been linked with fertility in cattle and yak (Hamilton et al., 2012; Zhang et al., 2019). (b) The *HSFY* family: Heat‐shock transcription factor Y‐linked (*HSFY*) is a member of the heat‐shock transcriptional factor (*HSF*) family that is found in multiple copies on the Y chromosome in many species. In cattle, this gene family is expanded compared to the human genome. It is worth noting that *HSFY* is dispersed along the goat Y chromosome, which is similar to cattle (Hamilton et al., 2011). (c) The *ZFY* family: *ZNF280AY* (zinc finger protein 280A, Y‐linked) and *ZNF280BY* (zinc finger protein 280B, Y‐linked) are lineage‐specific, multicopy Y‐linked gene families specific to cattle and derived from an autosome via a transposition to the Y chromosome (Yang, Chang et al., 2011; Yue et al., 2014). They have extensively expanded on the Y chromosome during evolution, and the copy number varies significantly among breeds and even individuals. These variations were associated with testis size and bull fertility (Yue et al., 2014). According to the multicopy phenomena we observed on goat Y, we assumed that these genes and families might reflect the evolutionary route of the goat Y chromosome.

### 3.4 The unique phylogenetic pattern of the goat Y chromosome

**FIGURE 3:**
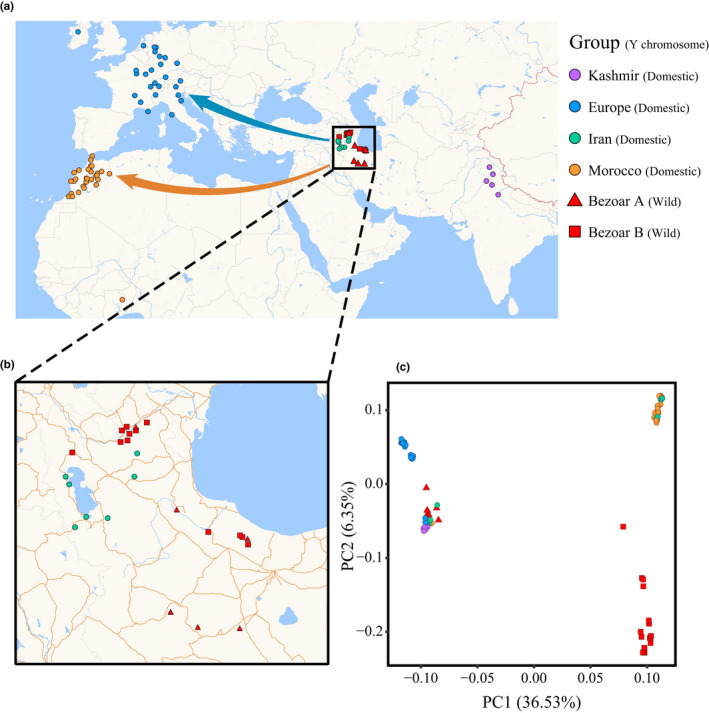
Population and relationship of goats based on the Y chromosome results. (a) The geographic map of 96 modern individuals, five outgroup samples were not shown due to the lack of geographic information. (b) The enlargement of the box in (a), exhibiting different distribution of two goat lineages, where lineage one is marked with a red triangle, and lineage two is marked with a red circle. (c) PCA constructed using Y SNP data. Colors reflect the geographic regions of sampling. Outgroup samples were not shown due to a high divergence from other goat samples

In total, 96 samples (distributed worldwide) were used for the phylogenetic analysis. This includes 24 bezoars from Northern and Central/Southern Zagros, 67 domestic goats from Europe, Morocco, Iran, and Kashmir (Figure 3a,b; Table S2), and five samples from other *Capra* species were set as outgroups. This included one *Capra falconeri*, two *Capra ibex,* and two *Capra sibirica*. The 33 scaffolds with length greater than 100 Kb were used as goat Y chromosome reference genome for SNP calling, and 6,914 high‐quality SNPs were kept for further analysis. The PCA plot showed that domestic goats and bezoars can be divided into two groups and that all 26 European and five Kashmiri domestic goats were closer to Bezoar A, while 24 Morocco domestic goats (28 in total) were closer to Bezoar B (Figure 3c). The Y phylogeny supported the deep divergence of two clades, Y1 and Y2, including both bezoars and domestic goats. The result does not support a previous proposition of a possible contribution of *Capra ibex* to domestic goats by hybridization with bezoars, producing the offspring possessing ibex‐type Y chromosome and bezoar‐type mtDNA (Pidancier et al., 2006). At least this possible hybridization pattern was not found in the areas where our samples were located.

**FIGURE 4:**
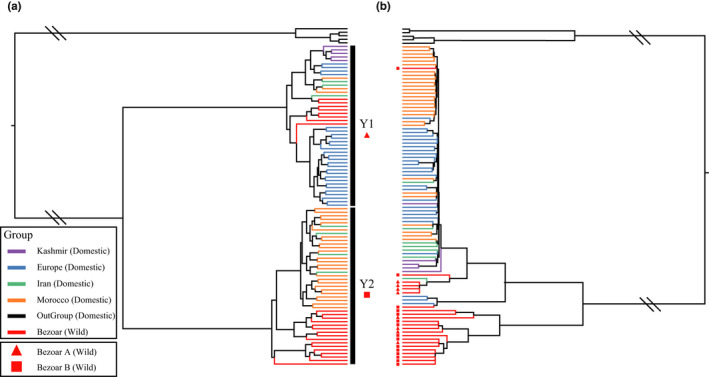
Phylogenetic tree of 96 modern goats. (a) Y chromosome haplotypes constructed using BEAST software. (b) mtDNA SNPs constructed using MEGAX software. Colors reflect the geographic regions of the samples

We then constructed phylogenetic trees using the Y chromosome, mitochondrial, and autosomal SNP information. The phylogeny from the Y chromosome differed from those inferred using autosomal and mtDNA data. The Y phylogeny divided bezoars and domestic goats into two clades (namely Y1 and Y2) (Figure 4a). This is consistent with the PCA results along the PC1. All domestic goats from Europe and Kashmir possessed the Y1 haplogroup, whereas domestic goats from Morocco were dominated by the Y2 haplogroup. Bezoars and domestic goats from Iran had both Y1 and Y2 haplogroups, with three samples belonging to Y1 and four samples belonging to Y2. The divergence of Y1 and Y2 predated the domestication of the goat. Haplotype network and phylogenetic tree of scaffold_213 data produced identical results, with two haplogroups found in goat populations, and unique geographical distribution among domestic goats (Figure S3). Besides, 1,771 SNPs showed significant variations between the two Y chromosome haplogroups, the majority was in the intronic or intergenic region. These SNPs corresponded to 75 gene regions, including testis‐specific Y‐encoded protein and heat‐shock transcription factor (Table S9). However, in the mtDNA phylogeny, most domestic goats formed one clade (A haplogroups), and there was no clear geographical structure among domestic goats in diverse regions (Figure 4b). The autosomal phylogeny revealed that all bezoars clustered into one clade and all domestic goats formed another clade (Figure S4). We further performed admixture analysis using autosomal data and found that regional goat populations were gradually separated as the number of components increased, except in Iran (Figure S5). Due to the possible effect from the uneven number of samples in different populations, we used expected geographic population distribution rather than cross‐validation values to explain admixture results.

To understand the demography of domestic goats, we generated a BSP. Using chromosome‐level complete sequences for BSP analysis is computationally resource‐intensive and time‐consuming. Two strategies were considered to improve the efficiency of the calculation: (a) Scaffold_213 and Scaffold_248 were selected to estimate the demographic history of the Y chromosome, but the results were different (Figure S6), suggesting variation information from 33 scaffolds would be better to approximate goat paternal demographic history; (b) we selected the concatenate SNPs and the full sequence of scaffold_213, respectively, for BSP analysis, and the results were consistent (Figure S7), indicating that the concatenate SNPs could replace the full sequence for BSP analysis. Moreover, this method had been applied in humans (Karmin et al., 2015). We further verified that the orders and orientations between different scaffolds did not affect the results (Figure S8), proving the validity and reliability of concatenating SNPs for BSP analysis.

**FIGURE 5:**
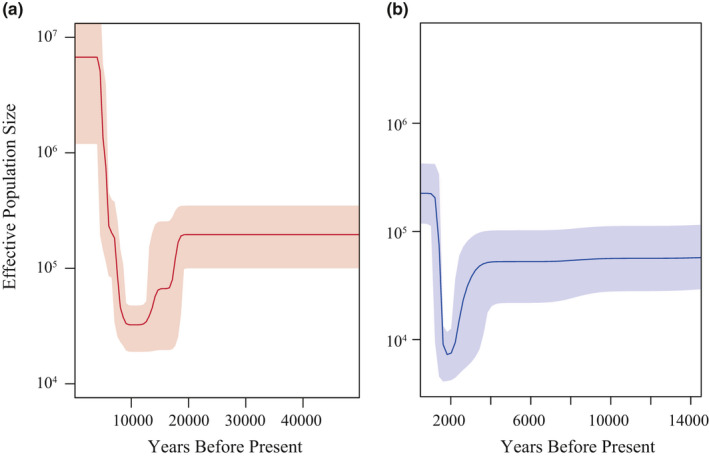
Comparison of Bayesian skyline plot for domestic goats using (a) Y SNP data (b) mtDNA sequence. The dark blue and red solid lines correspond to the Y chromosome and mtDNA median estimate of the effective population size (Ne). The shadow area shows the upper and lower limits of the estimation

From the Y chromosome information, goats experienced a population decline around 10,000 YBP (Figure 5a). In contrast, the mtDNA signal indicates that goat populations experienced a bottleneck around 2,000 YBP (Figure 5b). Therefore, the demographic history of goats shows apparent differences between the Y chromosome and mtDNA signals, which indicates unequal sex contribution during the domestication.

## 4 DISCUSSION

### 4.1 The assembly of the caprine Y chromosome sequence

In this study, the identification of candidate Y‐linked scaffolds was the basis for downstream scaffolding and assembling of the goat Y chromosome. According to the type and features of our data (short reads from NGS and long reads from PacBio), we applied an integrated strategy designed to produce an optimal balance between the sensitivity and accuracy. The YGS works well in identifying Y‐linked sequences, with a very low false‐positive error rate, both in a simple genome (like *Drosophila*) or in a complex genome (like human) (Carvalho & Clark, 2013). However, this method has low tolerance to single nucleotide differences, such as SNPs. Another challenge was that the candidate scaffolds were generated by PacBio sequencing and most of our data were Illumina‐generated. The innate sequencing bias between these two technologies limits the power of the YGS, especially in high‐GC and highly repetitive regions. We combined two other approaches in order to complement this result. AD ratio approach can reliably identify Y chromosome scaffolds based on the differences in average read depth between sexes. It can also be used to identify autosomes and X chromosome (Bidon et al., 2015). Compared with the YGS, this method has a higher tolerance to SNPs and the existence of different haplotypes, which helped us to identify Y‐linked scaffolds. Similarity search is based on the fact that both cattle and goat are ruminants and their general chromosomal structure is similar, so it can be used to identify Y‐linked scaffolds (Bickhart et al., 2017). Thus, we adopted above three approaches that have been applied in other projects with reliable results (Bidon et al., 2015; Hall et al., 2016) to identify Y‐linked scaffolds. We then combined different scaffolding strategies to generate the goat Y chromosome assembly, and the quality was improved significantly: (a) the N50 size of scaffolds rose from the original 63.5 Kb to 132.2 Kb in the draft assembly; (b) for Y‐linked scaffolds larger than 200 Kb, a subset of 5.9 Mb out of 8.7 Mb was successfully oriented and ordered; (c) the Y assembly was divided into two blocks: regions containing low‐copy genes and regions that harbor ampliconic genes.

We performed a tiered strategy integrating the RH map, Hi‐C, and mate‐pair library for extending and scaffolding. Hi‐C provides long‐range chromosome interaction information that decays rapidly as the linear distance within the chromosome increases. Thus, it is thought to be an unbiased approach that has been successfully applied in several genome projects (Burton et al., 2013; Lieberman‐Aiden et al., 2009; Putnam et al., 2016). However, long‐range DNA–DNA interactions may be too far apart for Hi‐C to directly ligate, and colocalization of different chromatins in the proximity of nuclear speckles may cause noises (Quinodoz et al., 2018). RH maps have been used extensively to improve and validate genome assemblies in many species (Du et al., 2014; Servin et al., 2012; Van Etten et al., 1999). Its resolution typically ranges from 50 Kb/cR to 1 Mb/cR and depends on the different radiation doses or hybrid numbers in the RH panel. Although each approach has its limitations, the integrated strategy proved capable of orchestrating independent information produced by each of these methods and generating a reliable Y assembly.

Previous cytogenetic studies have revealed that goat Y chromosome constitutes two parts: the p arm represents about half of the chromosome, containing the heterochromatic nucleolus organizer region (NOR), while the q arm, harboring the PAR (pseudoautosomal region) and the rest of the genome, is divided into a single copy and an ampliconic segment, respectively (Berardino et al., 2004; Di Meo et al., 2005; Schibler et al., 2009). Through the integration of the AD ratio, the YGS, and the similarity search, most Y‐linked scaffolds in the goat assembly could be reliably classified. In this study, we identified a total of 20.8 Mb of the goat Y chromosome, which is close to the typical size of the euchromatin part of mammalian Y chromosome (Li et al., 2013). Previous cytogenetic studies have revealed two features of the goat Y chromosome: (a) *ZFY* is located in low‐copy gene regions opposite *SRY* and on different arms; (b) the goat Y differs from the bovine Y in a pericentric inversion with a major loss of heterochromatin, which results in a smaller chromosome (Di Meo et al., 2005). In this study, we have generated the goat Y chromosome assembly, including the low‐copy gene region and the ampliconic (heterochromatic) region, through the integration of multiple scaffolding strategies. We found that almost all identified low‐copy genes were in the low‐copy region, except for *SRY*, which was situated on the scaffold_305, far away from this region (distal in the RH map). We also identified a total of 20.8 Mb candidate Y‐linked sequences. This supports the cytogenetic analysis result and further confirms that the caprine Y is smaller than the bovine Y (38.8 Mb) due to the loss of heterochromatin. Taken together, we have generated a reliable assembly of the goat Y chromosome, and further studies can rely on this draft for detailed exploration. Although the proposed assembly approaches still have difficulties, the tiered analysis represents a cost‐ and time‐effective in silico model of mammalian Y assembly.

### 4.2 The unique phylogenetic signal of the goat Y chromosome

Worldwide mtDNA analysis of caprine maternal lineages has revealed that the domestication of goat probably occurred independently in the Southern Zagros/Central Iranian Plateau and the Eastern Anatolia, with six different monophyletic mtDNA haplogroups (A, B, C, D, F, and G) identified (Luikart et al., 2001; Naderi et al., 2007). However, the A haplogroup represents >90% individuals of modern domestic goats (Naderi et al., 2008). Our mtDNA analysis results are consistent with these previous studies: most of the sampled domestic goat individuals belong to the A haplogroup and show a weak correlation between genetic distance and geographical distance. In contrast, the analysis of autosomal SNPs found that goat populations are highly structured, and genetic distance was strongly correlated with geographical distance in domestic goats (Colli et al., 2018; Rahmatalla et al., 2017). Besides, the bezoars and all domestic goats form two distinct monophyletic groups (Daly et al., 2018), which is consistent with our autosomal phylogenetic results. This geographic structure illustrates that goat populations underwent continuous selection, founder effects, and isolation during the domestication process. Many morphological traits of modern goats have been artificially selected over a long time, such as ear and horn shapes, coat colors, and wattles/long hair (Amills et al., 2017; Porter et al., 2016). Genes correlated with the nervous system, immunity, and productivity were also under selection (Alberto et al., 2018). Besides, with the development of human civilization, human preference for dairy products, cheese, and wool also increased in variety, which directly and indirectly additionally diversified the breeds of domestic goats (Amills et al., 2017).

Previous studies also used Y chromosome data to study the evolutionary history of paternal lineage, but due to scarcity of data, they only targeted a few specific SNPs, genes (*SRY, ZFY, AMELY*, and *DDX3YY*‐linked loci), or parts of the male‐specific region in the Y chromosome, to define the two major haplogroups (Cinar Kul et al., 2015; Nijman et al., 2020; Pidancier et al., 2006; Tabata et al., 2018; Vidal et al., 2017; Waki et al., 2015). The assembly of an almost complete Y chromosome allowed us to explore the Y‐linked genetic structure in much higher detail, improve our understanding of its internal structure, and helped us better understand the evolutionary history of goat domestication. The archeological results suggest that goats were first domesticated in the Fertile Crescent 10,000 YBP (Taberlet et al., 2008; Zeder & Hesse, 2000). However, domestication is usually seen as a long‐term process, which may involve the transformation from a hunter–gatherer civilization to a farming civilization (Colli et al., 2015). Our results showed that two highly divergent Y haplogroups exist in goat populations, each of which included both bezoars and domestic goats. Thus, we conclude that the origin of Y1 and Y2 haplogroups predates the domestication of goats. Besides, we found that two genes encoding heat‐shock transcription factor, six genes corresponding to testis‐specific Y‐encoded protein, and *SRY* gene showed significant differences between the two clades. These genes are involved in heat stress and development processes mainly regulated by the Y chromosome genes (Hansen, 2009).

The demographic history signals differed between the Y chromosome and mtDNA. The Y‐based analysis indicates that goats experienced a bottleneck around 10,000 YBP, around the time of the domestication of goat. mtDNA‐base analyses indicate that a decline in the effective population size occurred much later, around 2,000 YBP. The study of ancient samples revealed that after domestication goats dispersed in time and space around the Near East, and later goats bearing the A haplogroup spread worldwide after the Neolithic (Daly et al., 2018). Our BSP analysis of the mtDNA dataset confirmed that goats experienced a bottleneck around 2,000 YBP, which may be related to inbreeding caused by the human‐meditated livestock trade. However, the evidence from the Y chromosome reflects that male goats were not affected by this worldwide transmission in this period (Figure 5a), implying sexually unbalanced contribution in goat domestication. More specifically, female goats with desirable traits may have constituted a vast majority of the goat trade during the early trade period (2,000 YBP). This may have created a situation where cross‐breeding between native male goats and imported female goats (mainly bearing A haplogroup) was very common. The male offspring produced from these crosses would have possessed A haplogroup mitochondria and the native Y chromosome haplotype, which may explain the discordance between mtDNA and Y chromosome observed in our study.

## CONFLICT OF INTEREST

The authors declare no conflict of interest.

## AUTHOR CONTRIBUTION

**Changyi Xiao:** Formal analysis (equal); Visualization (equal); Writing‐original draft (equal); Writing‐review & editing (equal). **Jingjin Li:** Formal analysis (equal); Visualization (equal); Writing‐original draft (equal). **Hui Tang Xie:** Visualization (equal). **Hai Jian Chen:** Writing‐review & editing (equal). **Sijia Zhang:** Formal analysis (equal). **Salma Hassan Elaksher:** Formal analysis (equal). **Fan Jiang:** Formal analysis (equal). **Yaoxin Jiang:** Formal analysis (equal). **Lu Zhang:** Visualization (equal). **Wei Zhang:** Visualization (equal). **Yue Xiang:** Formal analysis (equal). **Zhenyang Wu:** Formal analysis (equal). **Shuhong Zhao:** Funding acquisition (equal); Writing‐review & editing (equal). **Xiaoyong Du:** Funding acquisition (equal); Writing‐review & editing (equal).

## ETHICS STATEMENT

This study was carried out in accordance with the guidelines of the science ethics committee of the Huazhong Agricultural University (HZAU).

## Supporting information

Fig S1‐S8

Table S1‐S9

## Data Availability

The Y genome assembly has been archived in the publicly accessible repository Dryad at https://doi.org/10.5061/dryad.bzkh1898c.
