# Supplementary material for: The assembly of caprine Y chromosome sequence reveals a unique paternal phylogenetic pattern and improves our understanding of the origin of domestic goat
Source: Ecol Evol. 2021 May 4;11(12):7779–95. doi: 10.1002/ece3.7611 (PMC8216945; doi:10.1002/ece3.7611)
Supplement: Supplementary file 1 — Fig S1‐S8 [file ECE3-11-7779-s001.docx]

**Supplementary Figures**


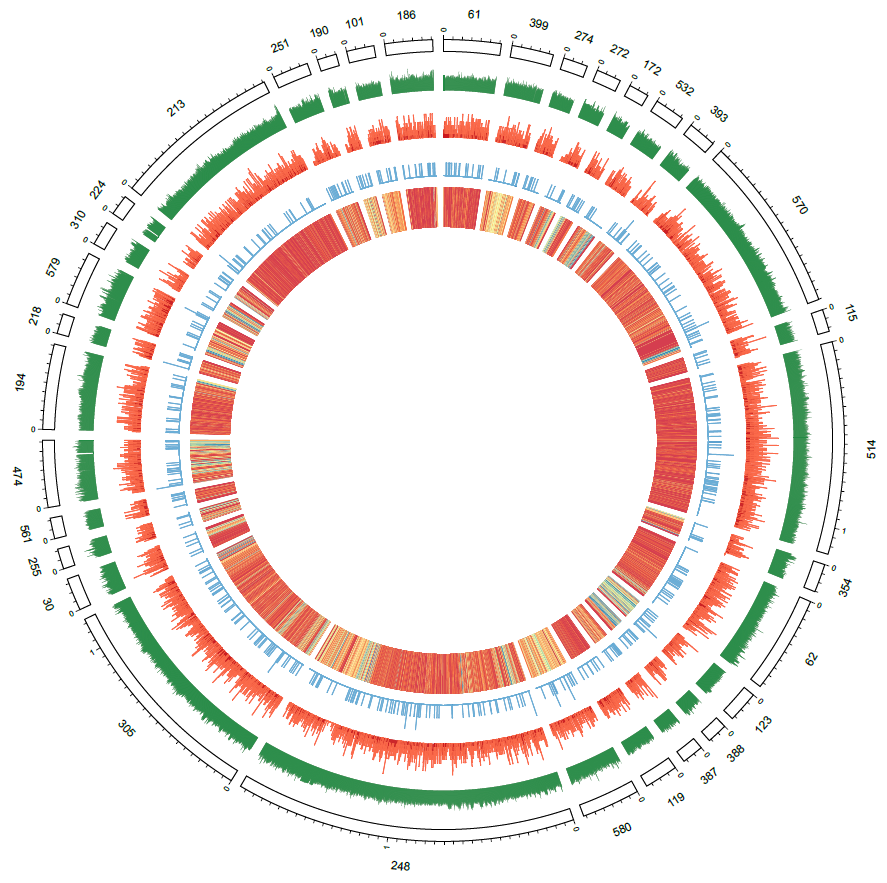


**Supplementary Figure 1.** Summary of 33 identified goat Y-linked scaffolds. From the outside to inside layer, (1) ideograms of 33 scaffolds (Mb); (2) GC content (%) of 1 Kb window; (3) the count of repetitive elements in a 1 Kb window; (4) the number of protein-coding genes in a 1 Kb window; (5) the density of SNPs called for each 1 Kb window.


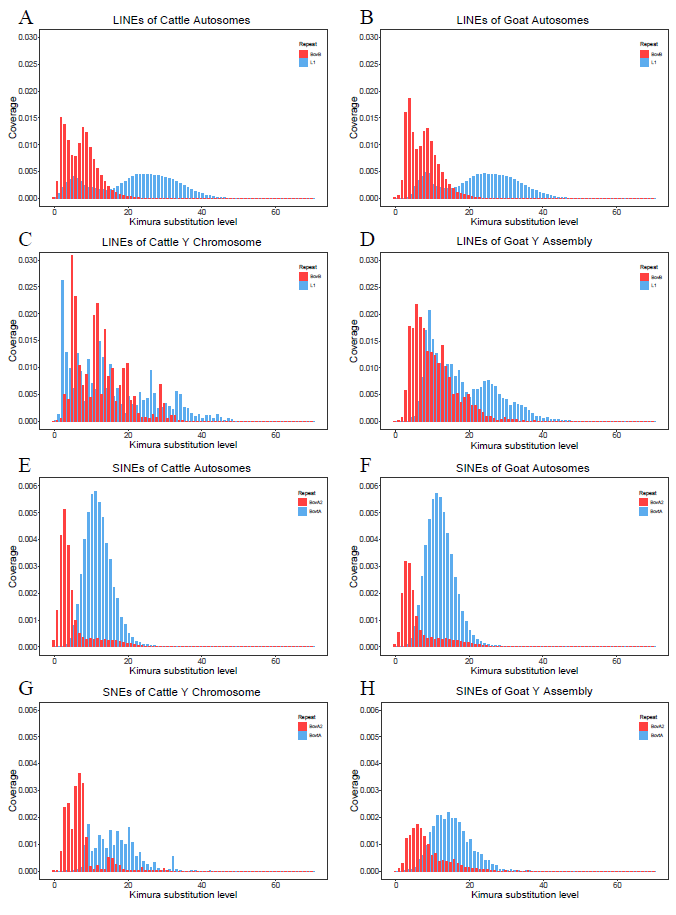


**Supplementary Figure 2.** Kimura divergence plot of BovB, L1, BovA2, and BovtA elements in the autosomes and Y chromosomes of cattle and goats. RepeatMasker was used to calculate the divergence. The y-axis represents coverage against the RepBase super consensus library; the x-axis indicates the Kimura divergence estimate.


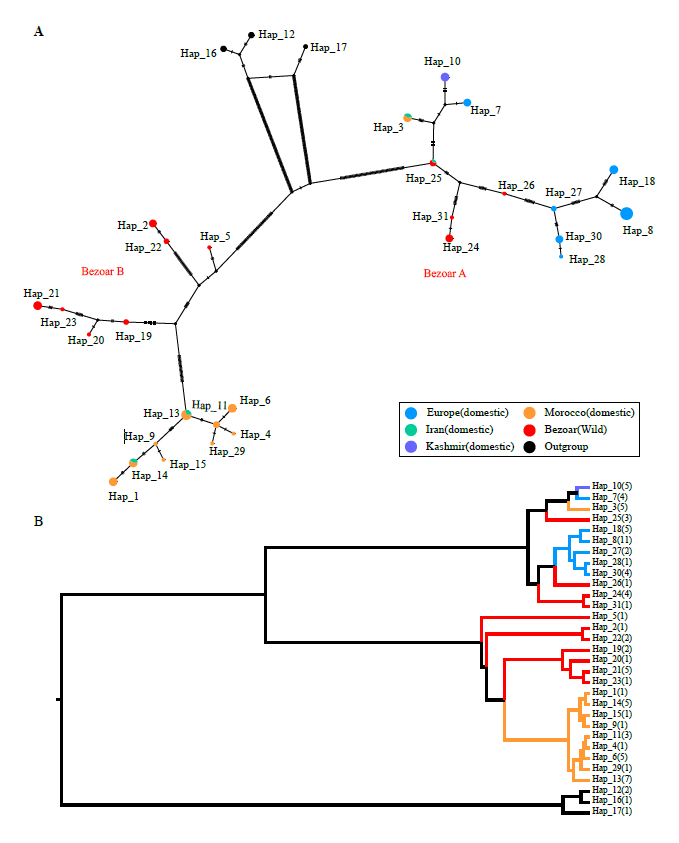


**Supplementary Figure 3.** **(A)** Haplotype network constructed using scaffold_213 full sequence data. (B) Phylogenetic tree of 31 haplotypes constructed using BEAST software.


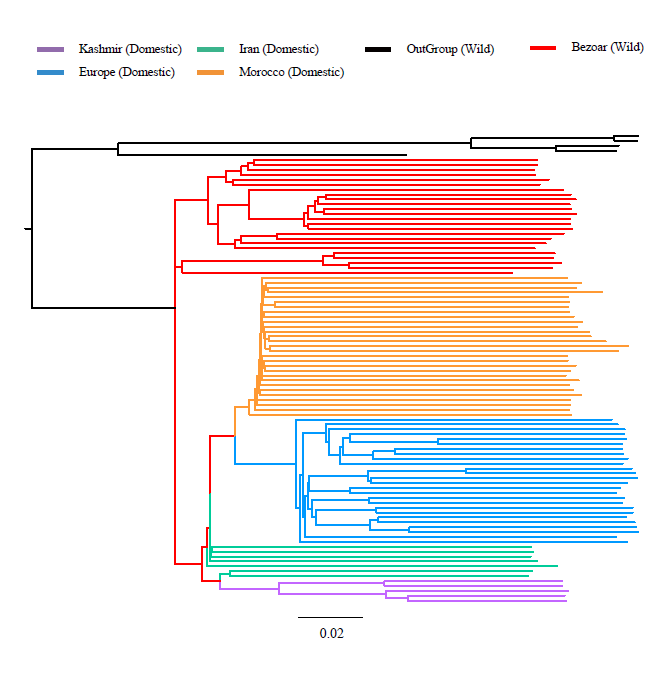


**Supplementary Figure 4.** NJ phylogenetic tree constructed using autosomal SNP data. Colors reflect the geographic regions of sampling.


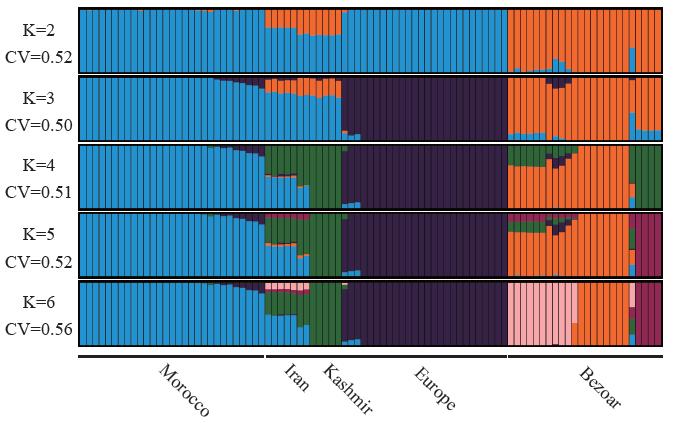


**Supplementary Figure 5.** Autosomal analysis for the origin of modern domestic goat. Model-based clustering of goat breeds using ADMIXTURE from K = 2 to K = 6. Breeds are separated according to geographic regions.


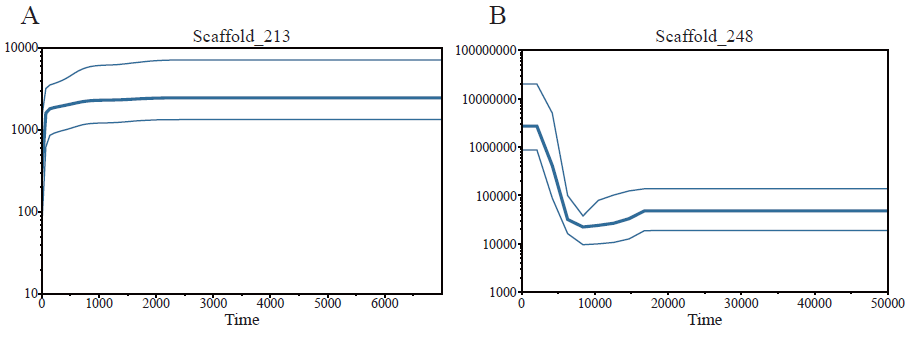


**Supplementary Figure 6.** Comparison of Bayesian Skyline Plot for domestic goats using different scaffold of the Y chromosome.


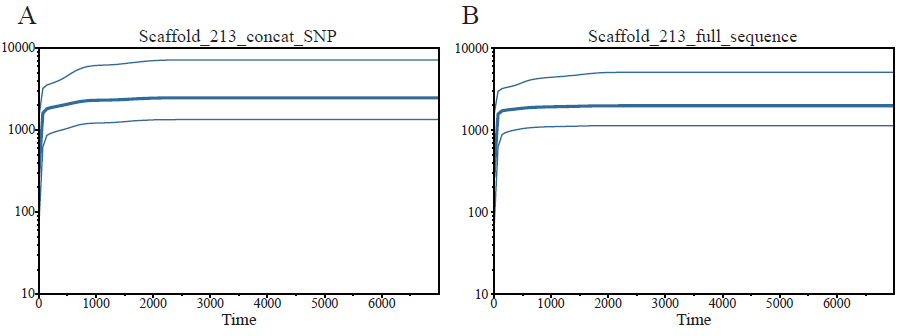


**Supplementary Figure 7.** Comparison of Bayesian Skyline Plot for domestic goats using (A)Y SNP data (B) full-sequence data of scaffold_213.


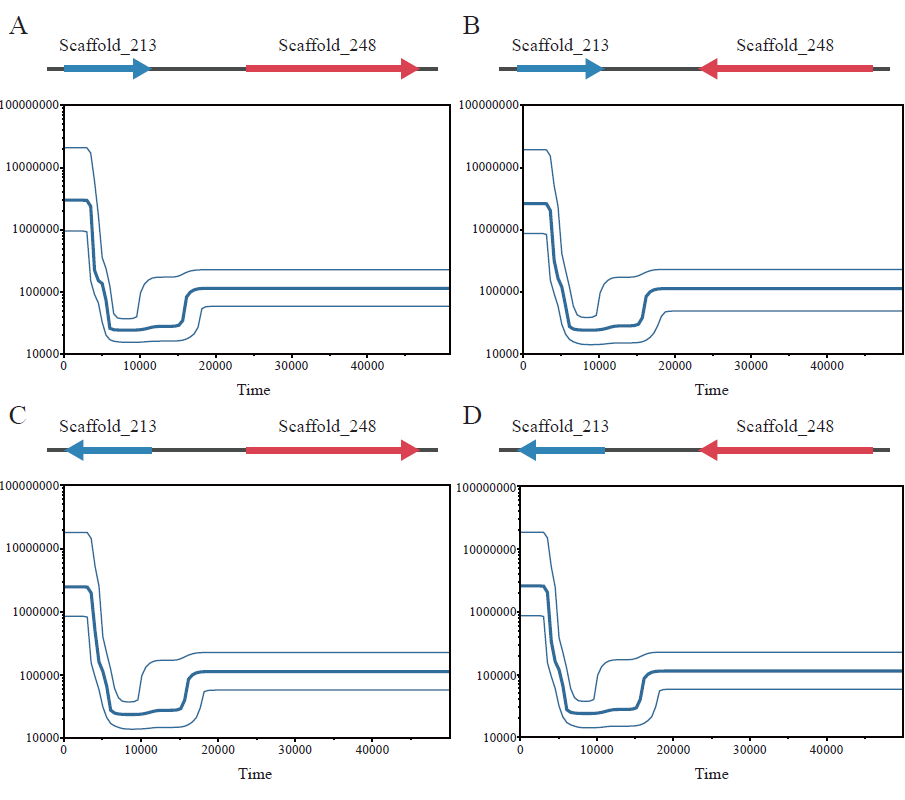


**Supplementary Figure 8.** Comparison of Bayesian Skyline Plot for domestic goats using different orders and orientations of the Y chromosome.
